# Functional and comparative genomics analyses of *pmp22 *in medaka fish

**DOI:** 10.1186/1471-2202-10-60

**Published:** 2009-06-17

**Authors:** Junji Itou, Mikita Suyama, Yukio Imamura, Tomonori Deguchi, Kazuhiro Fujimori, Shunsuke Yuba, Yutaka Kawarabayasi, Takashi Kawasaki

**Affiliations:** 1 Department of Radiation Biomedical Science IV, Radiation Biology Center, Graduate School of Medicine, Kyoto University, Yoshida-Konoe-cho, Sakyo-ku, Kyoto 606-8501, Japan; 2 Department of Genome Informatics, Center for Genomic Medicine, Graduate School of Medicine, Kyoto University, Yoshida-Konoe-cho, Sakyo-ku, Kyoto 606-8501, Japan; 3 Department of Molecular Oncology, Graduate School of Medicine, Kyoto University, Yoshida-Konoe-cho, Sakyo-ku, Kyoto 606-8501, Japan; 4 Tissue Engineering Research Group, Research Institute for Cell Engineering, National Institute of Advanced Industrial Science and Technology (AIST), 3-11-46 Nakoji, Amagamasaki, Hyogo 661-0974, Japan; 5 Genome Intelligence Research Group, Research Institute for Cell Engineering, National Institute of Advanced Industrial Science and Technology (AIST), 3-11-46 Nakoji, Amagamasaki, Hyogo 661-0974, Japan

## Abstract

**Background:**

*Pmp22*, a member of the junction protein family Claudin/EMP/PMP22, plays an important role in myelin formation. Increase of *pmp22 *transcription causes peripheral neuropathy, Charcot-Marie-Tooth disease type1A (CMT1A). The pathophysiological phenotype of CMT1A is aberrant axonal myelination which induces a reduction in nerve conduction velocity (NCV). Several CMT1A model rodents have been established by overexpressing *pmp22*. Thus, it is thought that *pmp22 *expression must be tightly regulated for correct myelin formation in mammals. Interestingly, the myelin sheath is also present in other jawed vertebrates. The purpose of this study is to analyze the evolutionary conservation of the association between *pmp22 *transcription level and vertebrate myelin formation, and to find the conserved non-coding sequences for *pmp22 *regulation by comparative genomics analyses between jawed fishes and mammals.

**Results:**

A transgenic *pmp22 *over-expression medaka fish line was established. The transgenic fish had approximately one fifth the peripheral NCV values of controls, and aberrant myelination of transgenic fish in the peripheral nerve system (PNS) was observed. We successfully confirmed that medaka fish *pmp22 *has the same exon-intron structure as mammals, and identified some known conserved regulatory motifs. Furthermore, we found novel conserved sequences in the first intron and 3'UTR.

**Conclusion:**

Medaka fish undergo abnormalities in the PNS when *pmp22 *transcription increases. This result indicates that an adequate *pmp22 *transcription level is necessary for correct myelination of jawed vertebrates. Comparison of *pmp22 *orthologs between distantly related species identifies evolutionary conserved sequences that contribute to precise regulation of *pmp22 *expression.

## Background

Peripheral myelination is important for rapid body movement and sensing several environmental stimuli [1]. The *pmp22 *gene encodes a hydrophobic tetraspan membrane protein which is a member of the junction protein family Claudin/EMP/PMP22 [2]. In the peripheral nerve system (PNS), PMP22 contributes to the formation and maintenance of the myelin sheath [3,4]. It is known that in mammals an increase of the *pmp22 *transcription level induces a reduction in nerve conduction velocity (NCV), coupled with abnormal axonal myelination. In Charcot-Marie-Tooth disease type1A (CMT1A) patients, the majority have a heterozygous tandem duplication of chromosome 17p11.2-p12, a 1.5 Mbp region that includes *pmp22*, and the patients undergo slow NCV and distal muscle weakness [3,5]. Thus, *pmp22 *transcription is likely to be tightly regulated to prevent over-expression. Understanding the regulatory mechanisms of this gene might improve treatment of CMT1A patients.

Promoter regions and some regulatory motifs of mammalian *pmp22 *have been reported. Transgenic rodent studies have shown the promoter regions and sequence conservation of the gene [6,7]. Some *in vitro *studies have identified regulatory motifs for Schwann cell specific expression of *pmp22 *[8,9]. However, unidentified functional sequences for modulation of *pmp22 *expression might be found by comparative genomics analyses.

The comparative genomics approach between jawed fishes and mammals facilitates the identification of novel conserved motifs [10,11]. Pufferfish, a teleost fish, have a compact genome, which has few redundant non-coding regions and is useful for finding functional sequences [12,13]. The elephant shark, a cartilaginous fish, has a genome with more conserved sequences with the human genome than the teleost fish genomes, and is expected to assist in the identification of novel essential motifs [14]. Pufferfish and the elephant shark are, however, unsuitable for experimental procedures. Two well known experimental teleost fish, zebrafish and medaka are available for both bioinformatics analyses and laboratory research.

In the present vertebrate phylogeny, the myelin sheath is seen only among jawed vertebrates [1,15]. It is unclear whether an adequate *pmp22 *transcription level is necessary for correct myelination in all jawed vertebrates. To address this issue, we established a transgenic medaka fish line overexpressing *pmp22 *and analyzed aberrations in their PNS. Although zebrafish is more universally used for experiments, we focused on medaka fish [16], because the medaka fish genome has shorter non-coding regions than the zebrafish [17], and thus is advantageous for bioinformatics analyses. Comparative genomics analyses were performed to identify conserved non-coding sequences. Our study reveals that *pmp22 *has an important role in myelin formation not only in mammals but also in fishes, and shows that the gene structure and some regulatory motifs are conserved among jawed vertebrates.

## Results

### *In silico *identification of the *pmp22 *ortholog in medaka fish

**Figure 1:**
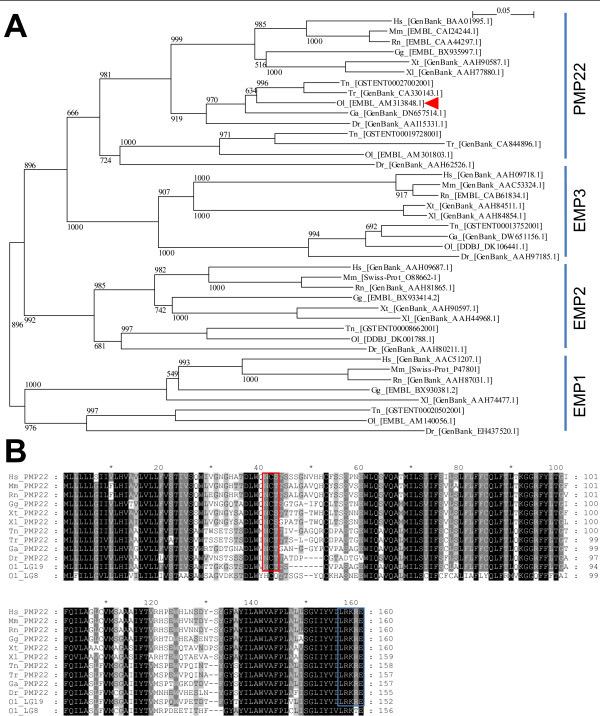
**Sequence conservation of PMP22 in jawed vertebrates**. (A) Phylogenic tree of amino acid sequences of the EMP/PMP22 family in jawed vertebrates was drawn by using clustelX and NJ plot. The numbers at the nodes are bootstrap values based on 10,000 replications. Sequences from each subfamily clustered together. The red arrowhead shows the corresponding sequence of *ol_pmp22*. (B) The amino acid alignment consisting of the mammalian, avian, amphibian, fish *pmp22 *genes and medaka fish paralogs was drawn by using clustelX and GeneDoc. The white letter on the black background indicates 100 percent conserved blocks. The white letter on the dark gray background indicates 80 percent or greater conserved blocks. The black letter on the bright gray background indicates 60 percent or greater conserved blocks. Red and blue squares indicate conservation of the N41 glycosylation site (NXS/T) and C terminus ER-retention/retrieval signal (LRKRE), respectively. Hs: *Homo sapiens *(human), Mm: *Mus musculus *(mouse), Rn: *Rattus norvegicus *(rat), Gg: *Gallus gallus *(chicken), Xt: *Xenopus tropicalis*, Xl: *Xenopus laevis*, Tn: *Tetraodon nigroviridis*, Tr: *Takifugu rubripes*, Ga: *Gasterosteus aculeatus *(stickleback), Ol: *Oryzias latipes *(medaka fish), Dr: *Danio rerio *(zebrafish).

To identify the *pmp22 *ortholog in medaka fish, a phylogenic tree of the EMP/PMP22 family was drawn based on the amino acid sequence alignment (Figure 1A). The teleost fish genomes, which have undergone another genome duplication compared to other vertebrates [12,18], have *pmp22 *paralogs. In medaka fish, two candidate EST sequences for *pmp22*, including the full length coding regions, are on LG8 and LG19, both of which correspond to human chromosome 17 [19]. The *pmp22 *cluster that includes mammalian orthologs is clearly separated from the other sequences (bootstrap value, 981). The medaka fish EST sequence AM313848, which maps to the syntenic region of LG19, clustered with mammalian PMP22 sequences. The transmembrane regions, N41 glycosylation site (NXS/T) and C terminus ER-retention/retrieval signal (LRKRE) [20] are conserved in the PMP22 cluster (Figure 1B). This suggests that the gene on LG19 has been conserved as *pmp22 *in medaka fish (*ol_pmp22*) and the other gene has been destined for degradation or neo-functionalization. Thus, we focused on the *ol_pmp22 *candidate encoded on LG19. *Ol_pmp22 *endogenous transcription was detected in multiple tissues of adult fish (additional file 1).

### Reduction in peripheral nerve conduction velocity and aberrant myelination in the *ol_pmp22 *over-expression fish

**Figure 2:**
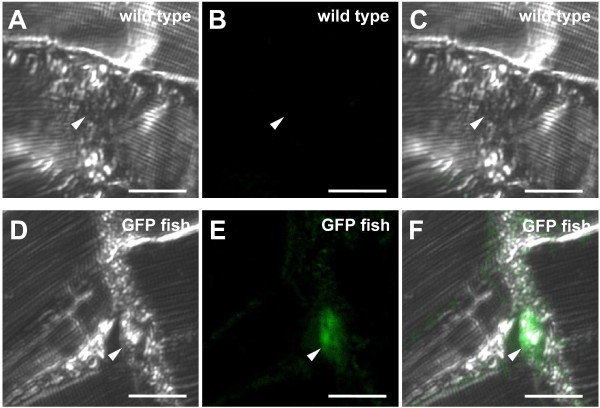
**GFP expression of the transgenic fish in adult Schwann cells**. Immunofluorescence staining of adult PNS was performed with a monoclonal antibody to GFP. Adult trunks of wild type (A-C) and transgenic GFP fish (D-F) were sliced horizontally (10 μm). The pictures show bright-field (A, D), fluorescence (B, E), and merged images (C, F). Myelin structures were observed between muscles (white arrow head). Bars: 20 μm.

**Figure 3:**
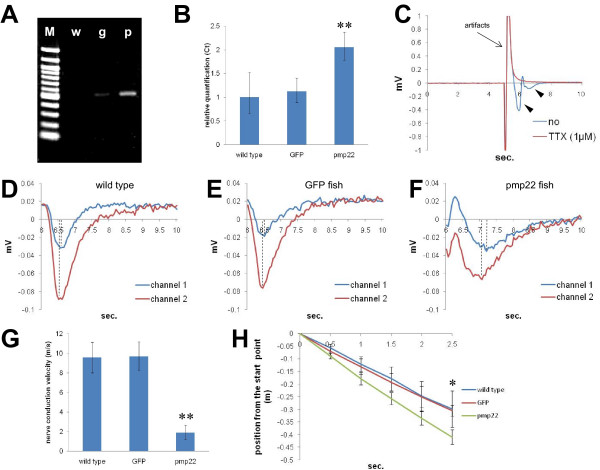
**Relation between *pmp22 *transcription levels and peripheral NCV, and swimming ability in medaka fish**. (A) The transgene, FLAG tagged *ol_pmp22*, expression in 8dpf fish. RT-PCR was performed with primers to FLAG and the SV40 polyadenylation site. M: 100 bp marker, w: wild type, g: GFP fish, p: pmp22 fish. (B) Relative transcription levels of *ol_pmp22 *in 8 dpf wild type, GFP fish and pmp22 fish. *Beta-actin *transcripts were used for an internal control. Relative transcription levels were quantified by defining the wild type average as 1.0 (0.656 to 1.522). Relative quantification of the GFP fish and pmp22 fish was 1.123 (0.898 to 1.405) and 2.056 (1.781 to 2.372), respectively. The minimum and maximum were estimated according to the manufacturer's instructions. Ct: threshold cycle. (**P < 0.05) (C) Population spikes stimulated by 100 μA with or without 1 μM TTX. The large spike is an artifact observed at around 5 seconds (black arrow). The spikes appearing at 6 to 7 seconds, action potential (black arrowhead), were not observed with TTX. (D, F, E) Population spikes of the wild type, D, GFP fish, E, and the pmp22 fish, F. Samples were stimulated with 60 μA. Spikes recorded at two adjacent channels (300 μm distance) were merged. The vertical dashed lines are drawn on each peak time. (G) Peripheral NCV was measured by dividing the distance between two channels by the time lag of the peaks. The peripheral NCV values of wild type, GFP fish and pmp22 fish were 9.566 ± 1.542 m/s, 9.700 ± 1.457 m/s and 1.924 ± 0.738 m/s, respectively (n = 5, **P < 0.05). (H) Flow distance of each line in running water (0.25 m/s). Positions from the start point was recorded every 0.5 seconds (n = 3, *P < 0.1).

We constructed a plasmid containing the promoterless FRT flanked EGFP coding sequence with a polyadenylation site. The FLP/FRT recombination system is more active than the Cre/loxP system at approximately 26°C [21], which is a suitable temperature for medaka fish breeding. An 11 kbp fragment containing the region upstream of the *ol_pmp22 *translation start codon and the FLAG tagged *ol_pmp22 *coding region with a polyadenylation site were inserted into the promoter and downstream of the FRT flanked region, respectively (additional file 2). This construct was injected into the one-cell stage medaka fish STII (wild type) [22]. We obtained a transgenic fish line with EGFP expression, regulated by the region upstream of the *ol_pmp22 *translation start codon, (GFP fish). We observed distinct GFP fluorescence from 3 day postfertilization (dpf) (additional file 3). Strong EGFP fluorescence was observed in the boundary region of the midbrain/hindbrain, longitudinal fissure of cerebrum, eye epithelium, olfactory epithelium, spinal cord, gill, liver, kidney, pharynx, intestine, bulbous arteriosus and fin fibroblast in the 10 dpf GFP fish (additional file 3). Though there was a time lag from GFP transcription to fluorescence, the GFP fish fluorescence pattern was consistent with a zebrafish *pmp22 *study, which was the first report of fish *pmp22 *[23]. An immunofluorescence study using anti-GFP antibody showed transgene expression in adult Schwann cells, indicating that the 11 kbp region upstream of the *ol_pmp22 *translation start codon contains sequences for Schwann cell expression (Figure 2). Then, we injected the synthesized *flp *recombinase mRNA into the one-cell stage GFP fish to remove the FRT flanked region, and established the *ol_pmp22 *over-expression fish line (pmp22 fish). The transgene, FLAG tagged *ol_pmp22*, was transcribed in the pmp22 fish, and transcribed at a low level in the GFP fish (Figure 3A). However quantitative RT-PCR showed no significant difference in total *ol_pmp22 *mRNA levels between the wild type and GFP fish. Compared to the control lines, wild type and GFP fish, the *ol_pmp22 *transcription level was approximately two-fold higher in the pmp22 fish (Figure 3B).

To observe the electrical excitability of the fish PNS, population spikes of nerve fibers activated by electrical stimulation were detected in sagittal sections of the adult fish trunk. Activated spikes were not observed with the sodium channel blocker, TTX (1 μM) with 100 μA stimulation, suggesting that the spikes were generated by electrical excitability of neurons (Figure 3C). We observed the peaks of population spikes immediately after 60 μA stimulation and estimated the NCV by the distance between two channels divided by the time lag of the peaks (Figure 3D, E, F). The wild type and GFP fish had similar NCVs (9.566 ± 1.542 m/s, n = 5 and 9.700 ± 1.457 m/s, n = 5, respectively), while the pmp22 fish had approximately one fifth the control line NCV value (1.924 ± 0.738 m/s, n = 5) (Figure 3G).

To test kinematic abnormalities, we observed medaka fish swimming in running water. Medaka fish usually swim against the current to keep their position. However, in a rapid current, the fish were swept downstream while swimming against the current. We measured flow distances of the wild type, GFP fish and pmp22 fish per time in rapid water current (0.25 m/s). The pmp22 fish had a tendency to be swept away faster than the control lines (Figure 3H), indicating that *pmp22 *over-expression causes weak swimming ability in medaka fish.

**Figure 4:**
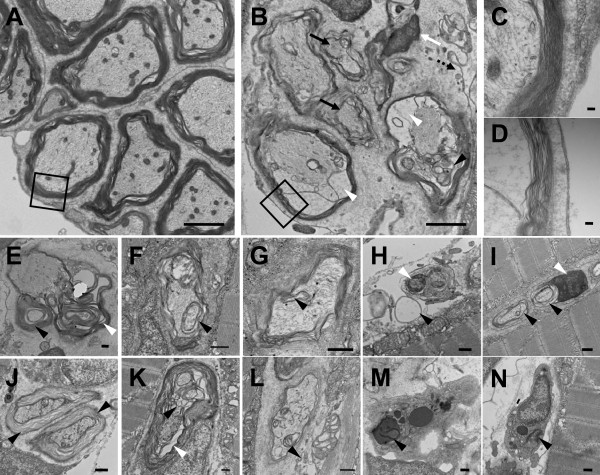
**Electron microscopy observation of cross sections of medaka fish motor nerve fibers**. Cross sections of the wild type (A, C) and the pmp22 fish (B, D-N) are shown. The pmp22 fish myelin sheath, D, was thinner than the wild type, C. The attenuated fiber (B; black arrow), enlarged nuclear (B; white arrow, H, I; white arrowhead), large periaxonal space (B, K; white arrowhead), granules (B, K; back arrowhead) and loose basal lamina (B, L; black dashed arrow) were observed in the pmp22 fish PNS. (E) Hypermyelination (black arrowhead) and outfolding (white arrowhead). (F, G) Infolding (black arrowhead). (H) Bare axon (black arrowhead). (I, J) Myelin uncompaction (black arrowhead). (M, N) Myelin debris (black arrowhead) was observed in macrophages. Bars: 2 μm for A and B, 100 nm for C and D, 500 nm for E-N.

From external observations, there were not obvious morphological and developmental abnormalities between the controls and pmp22 fish. However, aberrations were observed in cross sections of pmp22 fish motor nerve fibers by electron microscopy (Figure 4). The pmp22 fish might have undergone hypo- and hypermyelination in the PNS, similar to the mammalian CMT1A phenotype. In the pmp22 fish PNS, almost all observed myelin sheaths were likely to be thin and uncompacted (Figure 4D). We observed abnormal features, which looked like attenuated fibers, in- and outfolding myelin (Figure 4E, F, G), enlarged Schwann cell nuclei (Figure 4B, H, I), bare axons (Figure 4H), wide periaxonal spaces (Figure 4B, K), loose basal laminas (Figure 4B, L), and granules between the nerve fiber and myelin sheath (Figure 4B, K). Furthermore, we observed macrophages near the fibers with myelin debris (Figure 4M, N). These abnormalities were not observed in the wild type PNS. Although the typical onion bulb formation, which is the remarkable feature of demyelinating-remyelinating neuropathies, was not observed in the pmp22 fish, our observations indicate that prevention of *pmp22 *over-expression is important for keeping fast peripheral NCV and maintaining the myelin sheath in jawed vertebrates.

### Alternative first exons have been conserved in *ol_pmp22*

**Figure 5:**
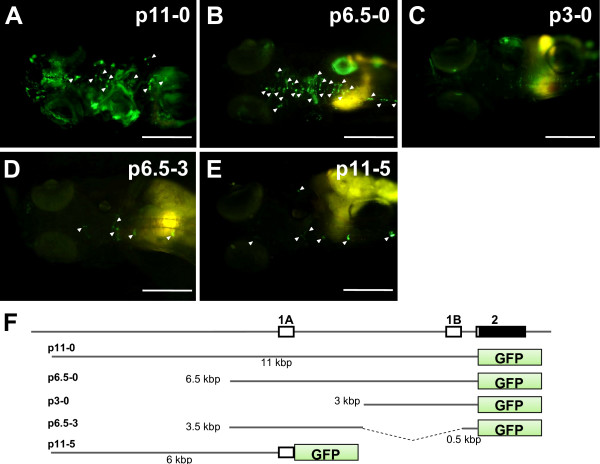
**GFP fluorescence in the transient expression assay for identification of the alternative first exons**. Transgene expression regulated by several constructs containing regions upstream of the translation start codon of *ol_pmp22 *was observed as GFP fluorescence. (A-E) Dosal view of anterior of fish with the p11-0, p6.5-0, p3-0, p6.5-3 and p11-5 constructs, respectively. White arrowheads show GFP expressing morphologically identified Schwann cells. Bars: 200 μm. (F) The alternative first exons and exon2 of *ol_pmp22 *and constructs for the transient expression assay.

Human and rodent *pmp22 *genes have alternative untranslated first exons, 1A and 1B [24]. Exon1A, located upstream of exon1B, is transcribed in Schwann cells, while the 1B transcript is ubiquitously expressed. In the database, *ol_pmp22 *EST sequences always have the same first exon. To confirm whether *ol_pmp22 *has a single first exon or alternative first exons, we constructed the EGFP expression constructs regulated by several regions upstream of the *ol_pmp22 *translation start codon, and analyzed the EGFP fluorescence patterns by a transient expression assay. The constructs, p11-0, p6.5-0, p3-0, p6.5-3, and p11-5, contain 11 kbp, 6.5 kbp, 3 kbp, 6.5 kbp to 3 kbp followed by 0.5 kbp, and 11 kbp to 5 kbp upstream of the translation start codon, respectively (Figure 5F). The fish injected with p11-0 and p6.5-0 had EGFP fluorescence in morphologically identified Schwann cells [25] and other tissues (Figure 5A, B). Although our transient expression assay was not quantitative, the p11-0 fish had more EGFP expression than the p6.5-0 fish, except in the Schwann cells. The p3-0 fish had no visible EGFP expression in the Schwann cells; however, it had fluorescence in other tissues (Figure 5C). Compared to the p3-0 fish, EGFP expression in the p6.5-3 fish was distinct in Schwann cells, but not in other tissues (Figure 5D). We identified the first exon of the EGFP transcript of the p6.5-3, as the region corresponding to *ol_pmp22 *exon1A (DDBJ: AB465505). The transcription start site (TSS) of the *ol_pmp22 *1A transcript is located 5,193 bp upstream of the translation start codon. Then we observed EGFP fluorescence only in Schwann cells of the p11-5 fish (Figure 5E). This finding suggests that the exon-intron structure of *pmp22 *has been conserved in jawed vertebrates.

**Figure 6:**
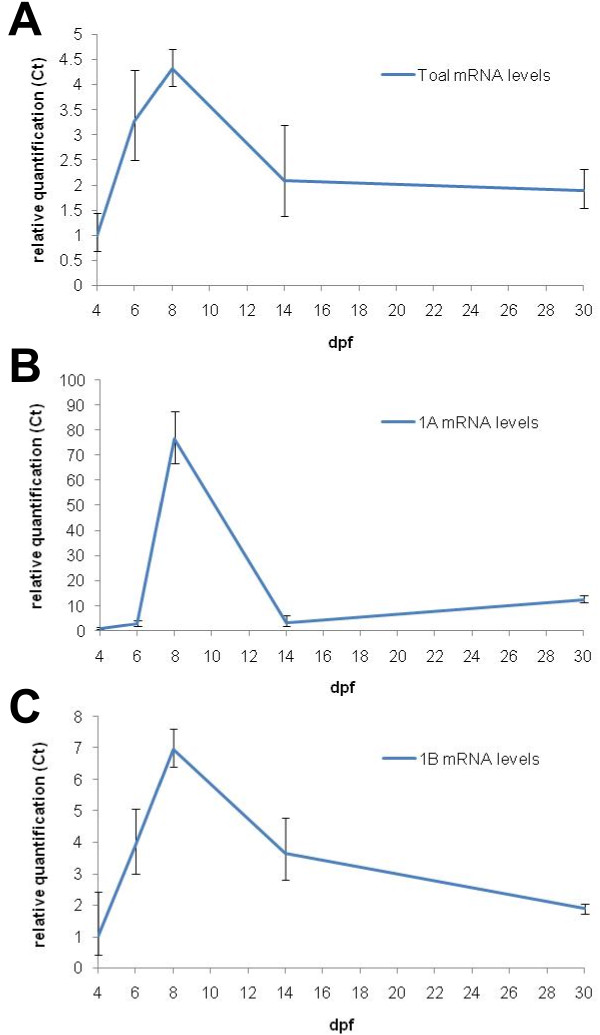
**Endogenous transcription levels of *ol_pmp22 *through medaka fish development**. Relative transcription levels of total (A), 1A (B) and 1B (C) are shown. Total RNA was prepared from mixed extract of some fish at 4, 6, 8, 14 and 30 dpf, and was used as template for quantitative RT-PCR. *Beta-actin *transcripts were used for an internal control. Each relative transcription level was quantified by defining the average of 4 dpf transcription level as 1.0. (P < 0.05).

Primer sets for the detection of 1A, 1B and total *ol_pmp22 *transcripts were designed. We measured mRNA levels in the whole bodies of 4, 6, 8, 14 and 30 dpf wild type fish (additional file 4). *Beta-actin *transcripts were detected for an internal control. Each average of 4 dpf *ol_pmp22 *levels was defined as 1.0 to quantify the relative transcription levels of total, 1A and 1B (Figure 6). The 1A transcript was significantly upregulated in 8 dpf, then decreased in 14 dpf and increased again in 30 dpf. On the other hand, in transient expression assays, the fish with p11-5, lacking the region downstream of exon1A, had no fluorescence until the hatching stage (8–10 dpf), whereas the others, p11, p6.5 and p6.5-3 fish, were visible in the Schwann cell lineage from the embryonic stage (data not shown). These results suggest that the regulatory mechanism of 1A transcription before the hatching stage is different from after hatching, and intron1A contains the region for embryonic stage specific regulation.

### Conserved sequences in the *pmp22 *region

Some regulatory motifs have already been identified in the promoter regions of human and rodent *pmp22 *exon1A [8,24]. NF1 is a conserved non-coding sequence of myelin genes and is located between the TATA-box and the TSS of the *pmp22 *1A promoter. SREB is the binding site for the sterol regulatory element binding proteins which are transcription factors for cholesterol homeostasis. The FP330 motif B is a conserved regulatory motif in myelin gene promoters. CREB, which is the binding site for the cyclic AMP response element binding protein, has been conserved in several gene promoters in vertebrates [26,27]. Thus, we analyzed the *pmp22 *conserved non-coding sequences in jawed vertebrates.

**Figure 7:**
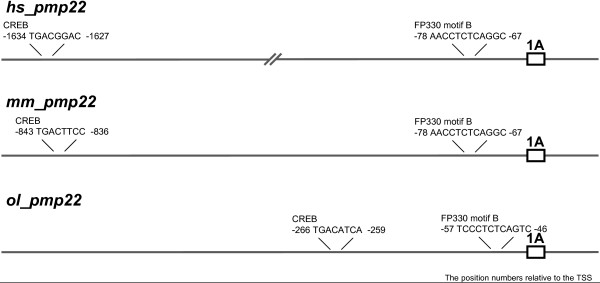
**Conserved motifs in the promoter region of exon1A of *hs_*, *mm_ *and *ol_pmp22***. The figure shows the positions of conserved regulatory motifs for *pmp22 *transcription. The numbers indicate the position relative to each TSS. The *hs_ *and *mm_pmp22 *have half of the CREB site (TGAC). The *ol_pmp22 *has the TG variant of CREB site (TGATGTCA). The position and sequence of the FP330 motif B (CTCTCAGGC) are conserved.

First, in the *ol_pmp22 *region, we examined the region upstream of exon1A and downstream of the stop codon (DDBJ: AB465503 and DDBJ: AB465504, respectively). We scanned for four known conserved mammalian motifs in the promoter region of *ol_pmp22 *exon1A by a motif finding program [28]. Although NF1 and SREB were not found, we identified two known regulatory motifs, FP330 motif B and CREB (Figure 7). The FP330 motif B is located immediately upstream of the TSS of human and rodent *pmp22 *exon1A [24]. The rat Schwannoma cell study revealed that the region around the FP330 motif B is involved with positive regulation of *pmp22 *1A transcription, and identified the important residues of this motif for DNA-protein interaction (CCAT), located at 3 bp to 6 bp downstream of the motif [9]. The FP330 motif B is also found in the promoter region of *ol_pmp22 *exon1A (-57 to -46, relative to the TSS of the *ol_pmp22 *1A transcript), however the important residues are not fully conserved (CAAA), which is the sequence at the corresponding position in mammals. In the promoter region of human *pmp22 *(*hs_pmp22*), CREB is identified as the silencer at 1.7 kbp upstream of the 1A TSS [8]. We identified CREB sites at -843 to -836 and -266 to -259 in mouse *pmp22 *(*mm_pmp22*) exon1A and *ol_pmp22 *exon1A, respectively.

**Figure 8:**
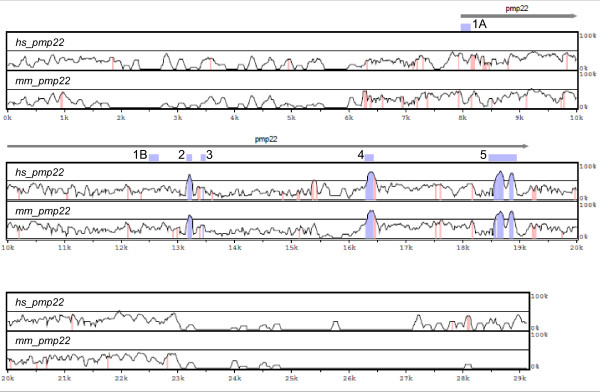
**Multiple alignment of the region of *pmp22 *in human, mouse and medaka fish**. The alignment was performed by LAGAN and presented by VISTA. The x-axis is the *ol_pmp22 *sequence. The y-axis is conservation from 0% to 100%. Top and bottom panels indicate the conservation of *hs_ *and *mm_pmp22 *with *ol_pmp22*, respectively. Blue and red shades indicate conservation, criteria: 70% or greater identity and minimum length of 20 bp, of transcribed regions (blue) and the other regions (red).

**Figure 9:**
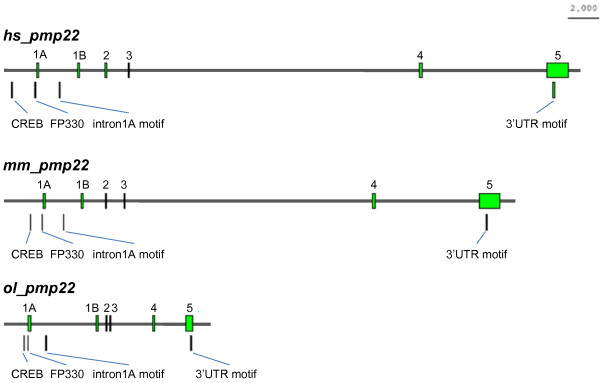
**Evolutionary conservation of the *pmp22 *gene structure and non-coding motifs**. The figure shows the structures of exons and conserved non-coding motifs (CREB, FP330, intron1A motif, and 3'UTR motif) of *hs_*, *mm_ *and *ol_pmp22*. Bar: 2000 bp.

**Figure 10:**
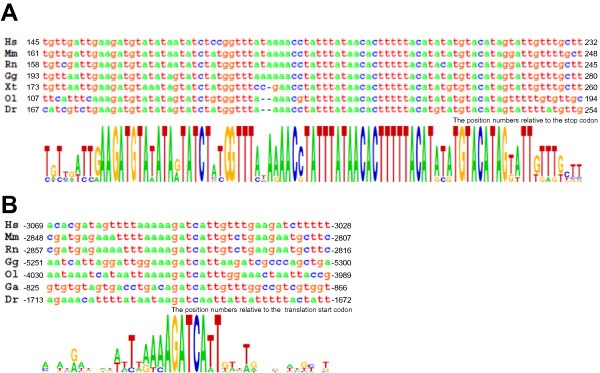
**Novel identified conserved sequences of *pmp22***. (A) Highly conserved sequences of the *pmp22 *3'UTR. The numbers indicate the position from each stop codon. (B) The conserved short sequences in the intron1A region of human, mouse, rat and medaka fish, and in the upstream region of exon1B of chicken, stickleback and zebrafish. The numbers indicate the position from each translation start codon. Sequence conservation is shown under the lines. Hs: *Homo sapiens *(human), Mm: *Mus musculus *(mouse), Rn: *Rattus norvegicus *(rat), Gg: *Gallus gallus *(chicken), Xt: *Xenopus tropicalis*, Xl: *Xenopus laevis*, Tn: *Tetraodon nigroviridis*, Tr: *Takifugu rubripes*, Ga: *Gasterosteus aculeatus *(stickleback), Ol: *Oryzias latipes *(medaka fish), Dr: *Danio rerio *(zebrafish).

To identify novel conserved non-coding sequences of *pmp22 *in jawed vertebrates, we performed comparative genomics analyses. Among *hs_*, *mm_ *and *ol_pmp22*, the multiple alignment of genomic sequences revealed some conserved sequences in the non-coding regions (Figure 8). Then, we analyzed these conserved regions in *pmp22 *of jawed vertebrates. Although, some conserved non-coding regions, located in intron2–4 among *hs_*, *mm_ *and *ol_pmp22*, were not found in other species, the 3'UTR sequence was highly conserved among jawed vertebrates, which includes an mRNA destabilization signal (ATTTA) (Figure 9, 10A). This conserved sequence starts approximately 100 bp to 200 bp downstream of the *pmp22 *stop codon. Next, we searched for short conserved sequences by a motif finding program [29]. We tried several combinations of the species in each region. One significant sequence pattern was found in intron1A among human, mouse, rat and medaka fish. Because exon1A has only been identified in human, mouse, rat and medaka fish, we searched for this pattern in the upstream region of exon1B in other species. This pattern was significantly conserved in jawed vertebrates, suggesting that the pattern is likely to be functional (Figure 9, 10B).

## Discussion

### *Pmp22 *over-expression causes reduction in peripheral NCV and aberrant myelination in jawed vertebrates

Increase of *pmp22 *expression causes CMT1A in humans and rodents. CMT1A patients, which have a heterozygous tandem duplication of a 1.5 Mbp region that includes *hs_pmp22*, have reduced peripheral NCV, aberrant myelination and distal muscle atrophy [3,5]. CMT1A rodent models with *pmp22 *over-expression, have been established to understand and treat this neuropathy [30-32]. In mouse models with a human YAC containing *hs_pmp22*, the degree of the CMT1A phenotype depends on the *hs_pmp22 *transcription level associated with YAC copy number [33,34]. The C22 mouse, a CMT1A model with 7 YAC copies, shows significant demyelinating neuropathy. A mouse study with tetracycline inducible *pmp22 *over-expression reveals that demyelination occurs only when *pmp22 *is highly expressed in adult, and that the neuropathic phenotype is reversible [35]. Rat *in vivo *and *in vitro *studies show that progesterone promotes *pmp22 *1A transcription in Schwann cells [36,37]. A CMT1A rat model, having 1.6 fold higher *pmp22 *transcription than wild type rats, is used for the progesterone antagonist test. The progesterone antagonist reduces *pmp22 *expression level. The treated CMT1A rats show higher motor performance than controls with the relief of muscle atrophy and sciatic nerve demyelination [38]. Ascorbic acid improves the CMT1A phenotype through the inhibition of cyclic AMP stimulation of *pmp22 *transcription. The ascorbic acid treated C22 mice have more activity, correct myelination and a longer life span than control neuropathy mice [39]. The principles of these treatments are based on the reduction of *pmp22 *transcription. Recently, another approach for the treatment of this neuropathy has been reported. In the C22 Schwann cells, there are large aggresomes, including PMP22 and ubiquitin, at perinuclear regions [40]. Also, the HSP90 inhibitor promotes heat shock protein expression, and corrects Schwann cell myelination with clearance of aggregated PMP22 [41].

The myelin sheath enables saltatory conduction in animals [1]. The jawed vertebrates have myelinated axons and conserved *pmp22*. To analyze the relationship between *pmp22 *transcription level and peripheral myelination in medaka fish, pmp22 fish were established. Compared with control lines, *ol_pmp22 *overexpressing fish, having approximately two-fold higher transcription of *ol_pmp22*, have one fifth peripheral NCV and aberrant PNS myelination, in spite of the transgene lacking intron 2–4 and the region downstream of the coding regions (Figure 3, 4). CMT1A patients and rodent models have hypo- and hypermyelination, attenuated fibers and onion bulb formations in the PNS [3,32]. In PNS, PMP22 interacts with α6β4 integrin to stabilize the attachment between the basal lamina and myelin sheath [42]. The β4 integrin and distroglycan double knockout mice lose myelin sheath stability with loose basal laminas and macrophage infiltration [43]. The macrophage-mediated myelin disruption occurs in human CMT1A with the *pmp22 *duplication [44], the C61 mouse with 4 YAC copies [45], the myelin glycoprotein P0 heterozygous deficient mouse [46], and the gap junction protein CX32 null mouse [47]. P0 and CX32 contribute to myelin formation in PNS [4]. Similar to these phenotypes observed in the PNS in mammals, we found loose basal laminas and macrophages including myelin debris in the PNS of pmp22 fish (Figure 4). Therefore our observations of the pmp22 fish suggest that the association of *pmp22 *expression levels with myelin maintenance is common among jawed vertebrates. Although, the pmp22 fish have normal growth, feeding and mating throughout life in the laboratory, it seems that prevention of *pmp22 *over-expression provides some advantages for selective pressure because of reduced NCV, weak swimming ability against the current and aberrant myelination caused by *pmp22 *over-expression in medaka fish.

### The exon-intron structure and non-coding motifs of *pmp22 *are conserved

The PMP22 sequence is highly conserved in jawed vertebrates, but less so in the chordate [48]. Alignments show that there are many conserved non-coding regions among mammals, and some regions in jawed vertebrates. Human and rodent *pmp22 *consist of alternative untranslated first exons, 1A and 1B [24], and four coding exons. Mammalian exon1A, located upstream of exon1B, is specifically expressed in Schwann cells. The alternative first exons also exist in *ol_pmp22*, suggesting that the common ancestor of teleost fishes and mammals had these exons, and the gene structure has been conserved in present jawed vertebrates. The *ol_pmp22 *1A transcript was upregulated in near hatching stage fish (Figure 6), which have immature Schwann cells with radial sorting in correspondence with zebrafish and rodent development [25,49-51]. Radial sorting is a step for differentiation from immature Schwann cells into myelinating Schwann cells. In this step, immature Schwann cells establish a 1:1 relationship with axons. On the other hand, as well as the rodent *pmp22 *1A transcript, which is elevated during myelination after birth [24,52], *ol_pmp22 *1A transcription increased after hatching (Figure 6). These results suggest that there is similar temporal expression of *pmp22 *1A transcription among jawed vertebrates.

Our bioinformatics analyses found that *ol_pmp22 *has two known conserved regulatory motifs (Figure 7). The FP330 motif B (CTCTCAGGC) is conserved immediately upstream of the TSS of *pmp22 *exon1A [9,24]. This motif is found in promoters of essential myelin genes in mammals. While in the promoter region of *hs_pmp22 *exon1A, a CREB half-site (TGAC) has been identified as the silencer responding to low cyclic AMP levels [8]. A CREB half-site and CREB TG variant (TGATGTCA) [27] were found in the *mm_ *and *ol_pmp22 *exon1A promoter region, respectively. However, it is unclear whether both of these CREB sites are functional.

In mammalian Schwann cells, *pmp22 *1A transcription is activated by the zinc finger transcription factor EGR2, which is the major positive regulator for essential myelin genes [53]. The direct target genes of EGR2 are *p0*, *mbp*, *mag*, *prx *and *dhh*, but not *pmp22 *[54,55]. In the *ol_pmp22 *region, the EGR2 binding motif was not found in our analyses. In a transgenic mouse study, over-expression of POU-domain protein POU3f1 downregulates *pmp22 *transcription [56]. There are many candidate sequences for POU3f1 binding in the promoter region of *ol_pmp22 *(data not shown); however it is unknown which sites are functional. MicroRNAs miR-9 and miR-29a inhibit *pmp22 *translation [57,58]. The binding sequences for miR-9 and miR-29a are conserved among human, rodent and chicken *pmp22*, but not among amphibian and jawed fish *pmp22*.

Furthermore, in this study, two additional conserved sequences were found. The *pmp22 *gene has highly conserved sequence in the 3'UTR, the length of which is about 70 bp, and includes an mRNA destabilization signal (Figure 10A). This 3'UTR sequence is unique in the genome. It is possible that the 3'UTR conservation contributes to modulating the PMP22 level by transcriptional or posttranscriptional regulation. By using a motif finding program, the novel conserved short sequence was found in intron1A of human, rodent and medaka fish *pmp22 *and in the upstream region of exon1B of the other species *pmp22 *(Figure 10B). In our transient expression assay, fish having the GFP expression construct, lacking the region downstream of exon1A, had no fluorescence until the hatching stage. These results suggest that the regulatory mechanism of 1A transcription in the embryonic stage is different from that after the hatching stage, and that this novel motif must be responsible for the embryonic stage expression of *pmp22*.

The DNA-binding domains of several transcription factors and short DNA sequences for transcription factor binding are usually conserved in animal genomes [59,60]. Comparison of the corresponding genome regions between fishes and mammals is useful for identification of functionally conserved sequences. We have successfully found novel conserved non-coding motifs (Figure 10), however further studies are necessary to fully understand *pmp22 *expression.

## Conclusion

We investigated the relation of *pmp22 *transcription level with PNS pathophysiology in medaka fish. Similar to the mammalian CMT1A phenotype caused by *pmp22 *over-expression, reduction in peripheral NCV and aberrant myelin formation were observed in the *pmp22 *over-expression fish, having two fold higher transcription of *ol_pmp22*. These results suggest that the contribution of adequate *pmp22 *expression in PNS is common among jawed vertebrates. Moreover, we elucidated the structural conservation of the gene among human, rodent and medaka fish, and found novel conserved non-coding motifs. These results indicate that the common ancestor for jawed vertebrates had acquired myelin formation with the associated gene functions including *pmp22 *and it has been conserved in present species.

## Methods

### Medaka fish breeding and selection

All fish lines were maintained at around 26°C under a 14 h light and 10 h dark cycle. Collected eggs were grown with 0.3% sea salt water. GFP transgenic fish were screened by fluorescent microscopy observation at embryonic and juvenile stages. Genotyping to select *pmp22 *over-expression fish was performed by PCR amplification of tail fin genomic DNA using primers ip166 (5'-CATGGATTACAAAGACGACGA-3') and ip167 (5'-ATGAGTCCGCTGATGAGCAC-3'). Manipulation of fish was performed following both national and institutional guidelines.

### DNA, RNA preparation and cDNA synthesis

Fish genomic DNA was prepared by phenol-chloroform and ethanol precipitation. Plasmids were prepared with the QIAprep Spin Miniprep Kit (Qiagen, Hilden, Germany). Total RNA was extracted by ISOGEN (Nippongene, Tokyo, Japan). First strand cDNA was synthesized by using the First-Strand cDNA Synthesis Kit (GE Healthcare, Tokyo, Japan).

### DNA construction

To construct the EGFP basic vector, an *NcoI*/*XbaI *digested EGFP coding fragment was obtained from a pEGFP vector (Clontech, Palo Alto, CA, USA) and inserted into the *NcoI*/*XbaI *digested pGL3-basic vector (Promega, Madison, WI, USA).

The 11 kbp region upstream of the translation start codon of *ol_pmp22 *was amplified by PCR with HdrR genomic DNA as the template and primers ip097 (5'-ATACGCGTATCATTTTCAAGGTCACTGGG-3') and ip098 (5'-AAGGATCCTTCACGGCCAGTCTAAGAAGAGAGGAGACA-3'). To construct the p11-0 EGFP expression vector, the amplified 11 kbp DNA fragment was digested with *MluI*/*BamHI*, and inserted into the *MluI*/*BglII *digested EGFP basic vector. The amplified 11 kbp DNA was digested with *BamHI *and partially digested with *BglII*, to obtain 6.5-0, 3-0 and 6.5-3 fragments. To construct the p6.5-0 and p3-0 vector, the fragments were inserted into the *BglII *digested and BAP treated EGFP basic vector. The 0.5 kbp fragment including the splice accepter sequence was obtained by PCR amplification of genomic DNA using primers ip119 (5'-TAAGATCTCTTTGCTCTGGGTTAAAGTT-3') and ip098, digested with *BglII*/*BamHI *and inserted into the *BglII *digested and BAP treated EGFP basic vector. To construct the p6.5-3 EGFP expression vector, the 6.5-3 fragment was inserted into the *BglII *digested and BAP treated EGFP vector containing the 0.5 kbp region upstream of the translation start codon. To construct p11-5, the 6 kbp fragment including *ol_pmp22 *exon1A and its upstream region was obtained by PCR amplification of genomic DNA using primer ip097 and ip134 (5'-TTTGGATCCAGTGAGGCTCTACCTGCTCG-3'), digested with *MluI*/*BamH*I and inserted into *MluI*/*BglII *digested EGFP basic vector.

The FRT flanked EGFP-BGHp(A) – FLAG-ol_pmp22 vector was constructed for establishing the *pmp22 *over-expression line. The FLAG-ol_pmp22 fragment was obtained by RT-PCR of medaka 8 dpf whole embryos using primers ip017 (5'-CCATGGATTACAAAGACGACGACGACAAAATGCTGATCTTA-3') and ip018 (5'-TCTAGATCATTCTCGCTTCCT-3'). The PCR product was digested with *NcoI*/*XbaI *and inserted into the *NcoI*/*XbaI *digested pGL3-basic vector. The fragment of BGHp(A) was amplified by PCR with phrGFPII-1 (Stratagene, La Jolla, CA, USA) as template and primers ip041 (5'-GGTCTAGATTCCCTTTAGTGAG-3') and ip042 (5'-GCGGATCCGGCGCCCCAGCATG-3'), digested with *XbaI*/*BamHI *and inserted into the *XbaI*/*BamHI *digested EGFP basic vector. To construct the FRT-flanked EGFP-BGHp(A) vector, the fragment was obtained by PCR amplification with the EGFP-BGHp(A) vector as template and primers ip044 (5'-CGAGATCTGAAGTTCCTATTCCGAAGTTCCTATTCTCTA GAAAGTATAGGAACTTCAAGCTTGGCATTCCGGTACTG-3') and ip045 (5'-AGAGCGCTCGAGGGCCCGTCTCCCATGAAGTTCCTATACTTTCTAGAGAA TAGGAACTTCGGAATAGGAACTTCGAATTCCGACGATAGTCATGCCC-3'), digested with *BglII*/*Eco47III *and inserted into the *BglII*/*Eco47III *digested pGL3-basic vector. An *NcoI*/*Eco47III *digested FLAG-ol_pmp22 fragment was obtained, and it was inserted into the *BsmBI*/*Eco47III *digested FRT-flanked EGFP-BGHp(A) vector. This vector was digested with *MluI*/*BglII *and ligated with the *MluI*/*BamHI *digested 11 kbp PCR product of the region upstream of the translation start codon (see additional file 2, the plasmid construct was drawn with PlasMapper [61]).

### Microinjection

Medaka fish fertilized eggs were collected immediately after spawning and placed into cold Yamamoto's solution (133 mM NaCl, 2.7 mM KCl, 2.1 mM CaCl_2_, 0.2 mM NaHCO_3_, pH 7.3). The solution (0.1 M K_3_PO_4_, 0.05% phenol red) with either plasmid DNA (30 ng/μl) or mRNA (400 ng/μl) was injected into the cytoplasm of one – cell stage eggs through the chorion. For *in vitro *capped mRNA synthesis, template DNA including the T7 promoter and FLP recombinase coding sequence were amplified by PCR with pOG44 as template and primers ip131 (5'-ATTAATACGACTCACTATAGGGCTCTCCACAGGTGTCCACTC-3') and ip132 (5'-ACCCGCACATACAGCTCACT-3'). *Flp *mRNA was synthesized by using the mMESSAGE mMACHINE T7 kit (Ambion, Austin, TX, USA) and polyadenylated by the Poly (A) Tailing kit (Ambion). A pressure injector, FemtoJet (Eppendorf, Hamburg, Germany), was used with borosilicate glass capillaries GC100F-10 (Harvard Apparatus, Kent, UK).

### Immunofluorescence

Adult trunks were removed along with the bones and fixed in 4% paraformaldehyde in phosphate buffered saline (PBS) (pH 7.4) at 4°C for several hours. Fixed samples were decalcificated by 6% sucrose at 4°C overnight. Infiltration and embedding in glycol methacrylate were performed with Technovit 8100 (Heraeus Kulzer, Werheim, Germany). Embedded samples were sliced horizontally (10 μm) by a rotary microtome, Leica RM2165 (Leica, Nussloch, Germany) equipped with a disposable microtome blade N35 (Feather, Osaka, Japan), stretched with distilled water on slide glasses, and dried at room temperature (RT) overnight. After soaking with PBS, samples were incubated with 0.1 mg/ml proteinase K at 37°C for 15 min, rinsed with PBS, incubated with blocking solution (1% horse serum in PBS with 0.5% Tween 20) at RT for 1 h, incubated with 1/500 anti-GFP monoclonal antibody (Nacalai tesque, Kyoto, Japan) in blocking solution at 4°C overnight, rinsed with PBS with 0.5% Tween 20, incubated with 1/500 Alexa fluor 488 conjugated anti-mouse IgG antibody (Invitrogen, Eugene, OR, USA) in blocking solution at RT for 1 h, and rinsed with PBS. Bright-field and fluorescence images were observed using a confocal microscope FV1000 (Olympus, Tokyo, Japan) and data were obtained by Fluoview ver 1.6b software (Olympus).

### 5'-RACE

Total RNA was prepared from whole bodies of p6.5-3 injected fish at 14 dpf. The SMART RACE cDNA Amplification kit (Clontech) and SuperScript II Reverse Transcriptase (Invitrogen) were used for first strand cDNA synthesis. RACE PCR was performed by using the Advantage 2 PCR kit (Clontech) with primer ip128 (5'-TAGGTCAGGGTGGTCACGAGGGT-3').

### Quantitative RT-PCR

The target mRNA quantification was performed by using the Power SYBR Green PCR Master Mix (Applied Biosystems, Foster City, CA, USA). The following primers were used: ip150 (5'-GCTCTCTGATGATTCTTGCTTTTG-3') and ip151 (5'-AGGAGCAGAATGTCCAGAATGC-3') for the 1A transcript, ip152 (5'-CTACTGCAGGTCCACTCTTTTGG-3') and ip153 (5'-GGCCAGTTTCAAGCAAATCG-3') for the 1B transcript, ip160 (5'-TCCTCTTCTTCTGTCAGCTCTTCA-3') and ip161 (5'-GCTCCGCTCATCACAAACAA-3') for total *ol_pmp22 *transcript, ip154 (5'-GCCCCACCAGAGCGTAAATAC-3') and ip155 (5'-CATCGTACTCCTGCTTGCTGAT-3') for *beta-actin*[62]. Relative quantification was calculated according to the manufacturer's instructions.

### Electrophysiology

The electrical excitability of fish nerve was measured according to Imamura Y., 2008 [63]. Sagittal fish slices (500 μm) were cut by a vibrating microtome, DTK-1000 (DSK, Kyoto, Japan) in an oxygenated (95% O_2_/5% CO_2_) physiological solution, artificial cerebrospinal fluid (ACSF) at 4°C, containing: 126 mM NaCl, 2.5 mM KCl, 1 mM MgCl_2_, 26 mM NaHCO_3_, 1,25 mM NaH_2_PO_4_, 2 mM CaCl_2 _and 10 mM D-glucose. The sample was attached to a 64 channel-electrode dish perfused with ACSF (3 ml/min). Peripheral nerve fibers were stimulated with 0.1 Hz, 60 or 100 μA, and population spikes of fish nerve fibers at 300 μm, 600 μm, and 900 μm distance between two channels were recorded in the perfusion of ACSF. The recording was stored in Windows XP hardware and data analysis was performed with Mobius software of the MED64 system (Alpha MED Sciences, Osaka, Japan). Statistical analysis was performed by Student's t-test.

### Swimming ability test

The medaka fish swimming ability test was performed in running water (0.25 m/s) in a straight horizontal watercourse (depth 30 mm, width 70 mm, length 1 m) at room temperature. Adult male fish were used. The volume of water flow from a 15 mm diameter hose was 188 ml per second. Medaka fish were put into a decelerated water current in a net for 30 seconds and gently released into the rapid water current. The movie of a top view of fish swimming was acquired by a digital video camera, NV-GS250-S (Matsushita, Osaka, Japan). A picture of each time point was obtained by MotionDV STUDIO 5.3J LE for DV software (Matsushita), and analyzed by the scion image software (Scion, Frederick, MD, USA). Statistical analysis was performed by Student's t-test.

### Electron microscopy

Adult trunks were removed along with the bones and cut into 1–2 mm pieces. Samples were fixed in 2% glutaraldehyde in 0.1 M phosphate buffer (pH 7.4) for 2 h, washed in 0.1 M phosphate buffer (pH 7.4) for 20 min 5 times and postfixed in 1% OsO_4 _in phosphate buffer (pH 7.4) for 2 h. Then samples were dehydrated: 50% ethanol for 10 min, 60% ethanol for 10 min, 70% ethanol for 10 min, 80% ethanol for 10 min, 90% ethanol for 10 min, 99% ethanol for 10 min and 100% ethanol for 20 min twice. Dehydrated samples were transferred to propylene-oxide for 20 min twice, 1:1 propylene-oxide:epoxyresin for 1 h, 1:3 propylene-oxide:epoxyresin for 1 h and epoxyresin overnight. For embedding, samples were left at 60°C with fresh epoxyresin three overnight. Ultra thin sections (80 nm) were obtained on an Ultra microtome EM UC6 (Leica, Vienna, Austria), counterstained by Reynolds method and examined on an H-7650 (Hitachi, Tokyo, Japan). Medaka fish motor nerve fibers were determined on the locations according to the studies for zebrafish motor nerve fibers [64,65].

### Bioinformatics analysis

Sequences of the EMP/PMP22 family were obtained by BLAST and BLAT, using human amino acid sequences of the family as the queries. For phylogenic analysis, mRNA sequences were translated into amino acid sequences. The analyses for conserved motifs were performed by the scan for matches [28], Weeder [29] and jasper [66]. Multiple alignment of *pmp22 *was performed by LAGAN [67] and presented by VISTA [68].

## List of abbreviations

*pmp22*: peripheral myelin protein 22; CMT1A: Charcot-Marie-Tooth disease type1A; NCV: nerve conduction velocity; PNS: peripheral nerve system; EMP: epithelial membrane protein; UTR: untranslated region; LG: linkage group; FRT: FLP recognition target; EGFP: enhanced green fluorescent protein; dpf: day postfertilization; TTX: tetrodotoxin; EST: expression sequence tags; TSS: transcription start site; CREB: cyclic AMP response element binding; YAC: yeast artificial chromosome; ACSF: artificial cerebrospinal fluid; PCR: polymerase chain reaction.

## Authors' contributions

JI carried out the transgenic, molecular, electron microscopic, kinematic and bioinformatics studies; and participated in the design and drafted the manuscript. MS carried out the bioinformatics studies, participated in the design and helped to draft the manuscript. YI carried out the electrophysiological studies. TD and KF carried out the molecular studies and participated in the design. SY and YK participated in the design and coordination. TK carried out the molecular and kinematic studies, participated in the design and helped to draft the manuscript. All authors read and approved the final manuscript.

## Supplementary Material

Additional file 1 **Endogenous expression of ol_pmp22 in adult fish**. Total RNA prepared form several tissues of adult fish was used as template for RT-PCR (30 cycles of 94°C for 15 sec, 60°C for 30 sec and 68°C for 30 sec). The primers were ip008 (5'-GGAATCATCCTGCTGCACAT-3') and ip009 (5'-GGGTTGCAGTTAAGGTTACCG-3'). ey: eye, br: brain, li: liver, in: intestine, pa: pancreas, ga: gallbladder, ki: kidney, he: heart, fi: fin, ov: ovary, te: testis, M: 100 bp marker. Click here for file

Additional file 2 **The construct for establishing the pmp22 fish**. Amplified FRT flanked EGFP with a BGH polyadenylation site was digested with *BglII*/*Eco47III *and inserted into the *BglII*/*Eco47III *digested pGL3-basic vector (pFRT-EGFP-BGHp(A)-FRT). The 11 kbp region upstream of *ol_pmp22 *translation start codon was inserted into the plasmid digested with *MluI*/*BglII*. The FLAG tagged ol_pmp22 coding sequence with an SV40 polyadenylation site was inserted into the plasmid digested with *BsmBI*/*Eco47III*. Click here for file

Additional file 3 **GFP fluorescence of GFP fish**. The pictures show fluorescent microscopy observations, A-O, and light microscopy, A'-O', of GFP fish. Embryonic stages, 0 dpf (A, A'), 1 dpf (B, B'), 2 dpf (C, C'), 3 dpf (D, D'), 4 dpf (E, I, J, E', I', J'), 5 dpf (F, K, F', K'), 6 dpf (G, G') and 7 dpf (H, H'), are shown. Distinct fluorescence was observed from 3 dpf. In embryonic stages, strong GFP fluorescence was observed in nervous system, branchial arches (I; white arrow) and olfactory epithelium (J, K; white arrow). Dorsal view of the anterior (L, L'), left side view of the middle, (M, M'), left side view of the tail (N, N'), and ventral view of the anterior (O, O') of 10 dpf are shown. ey: eye, mb: midbrain, hb: hindbrain, sp: spinal cord, lsc: longitudinal fissure of cerebrum, li: liver, ki: kidney, in: intestine, yo: yolk, fi: fin fibroblast, ba: bulbous arteriosus, gi: gill, ph: pharynx. Bars: 200 μm. Click here for file

Additional file 4 **Values of mRNA quantification of the total, 1A and 1B transcription levels**. Total RNA was prepared from mixed extract of some fish at 4, 6, 8, 14 and 30 dpf. The upper table shows the threshold cycles of the *beta-actin*, internal control, and *ol_pmp22 *mRNA. The lower table shows the relative quantification of total, 1A and 1B transcript defining the average of each 4 dpf transcription level as 1.0. The graph is shown in Figure 6. The minimum and maximum were estimated according to the manufacturer's instructions (P < 0.05). Click here for file
