# Supplementary figures and images for: Functional and comparative genomics analyses of pmp22 in medaka fish
Source: BMC Neurosci. 2009 Jun 17;10:60. doi: 10.1186/1471-2202-10-60 (PMC2714311; doi:10.1186/1471-2202-10-60)

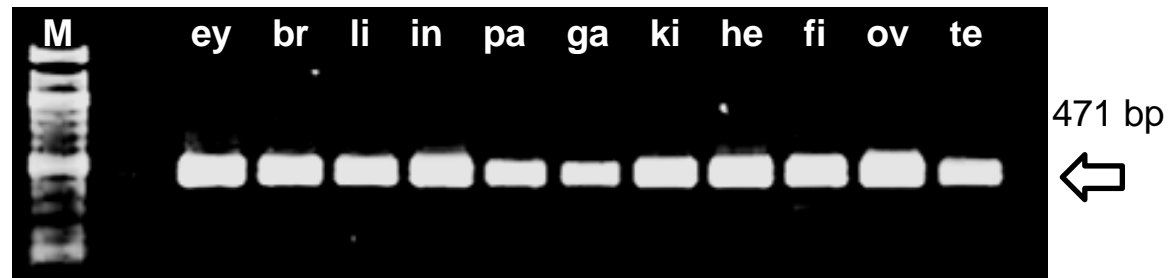

Supplement: Additional file 1 — Endogenous expression of ol_pmp22 in adult fish. Total RNA prepared form several tissues of adult fish was used as template for RT-PCR (30 cycles of 94°C for 15 sec, 60°C for 30 sec and 68°C for 30 sec). The primers were ip008 (5'-GGAATCATCCTGCTGCACAT-3') and ip009 (5'-GGGTTGCAGTTAAGGTTACCG-3'). ey: eye, br: brain, li: liver, in: intestine, pa: pancreas, ga: gallbladder, ki: kidney, he: heart, fi: fin, ov: ovary, te: testis, M: 100 bp marker. [file 1471-2202-10-60-S1.pdf]

upstream region of translation start codon of *ol\_pmp22*

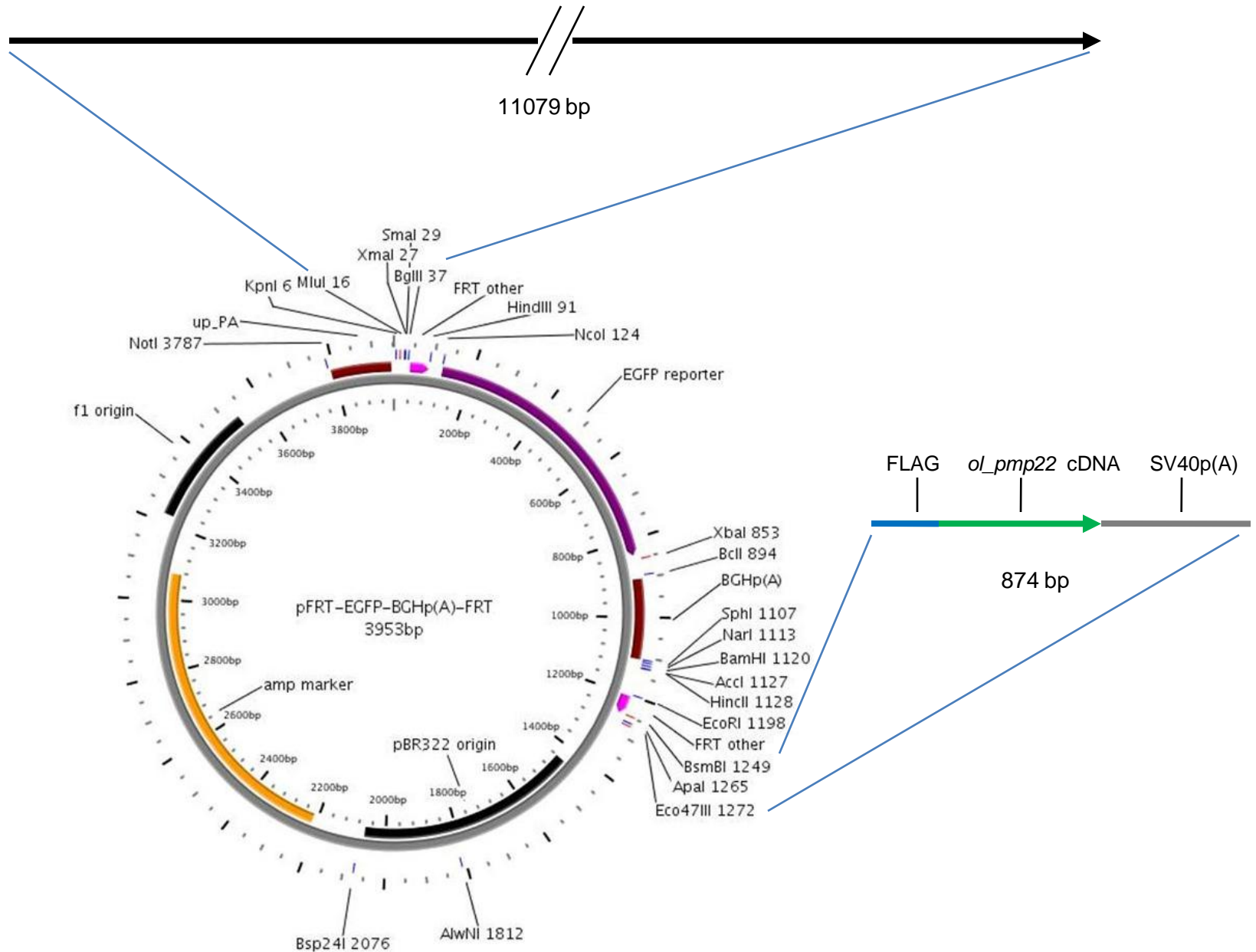

Supplement: Additional file 2 — The construct for establishing the pmp22 fish. Amplified FRT flanked EGFP with a BGH polyadenylation site was digested with BglII/Eco47III and inserted into the BglII/Eco47III digested pGL3-basic vector (pFRT-EGFP-BGHp(A)-FRT). The 11 kbp region upstream of ol_pmp22 translation start codon was inserted into the plasmid digested with MluI/BglII. The FLAG tagged ol_pmp22 coding sequence with an SV40 polyadenylation site was inserted into the plasmid digested with BsmBI/Eco47III. [file 1471-2202-10-60-S2.pdf]

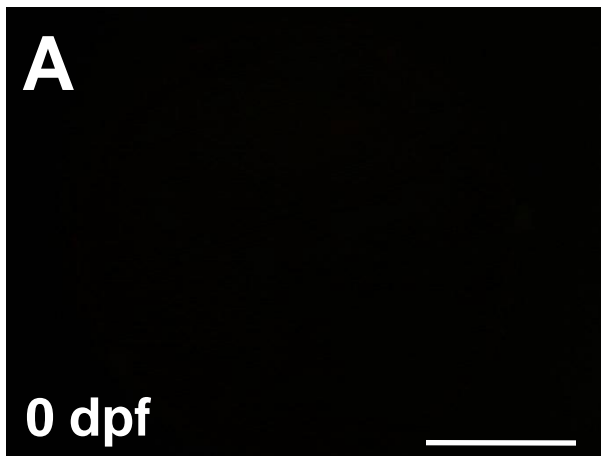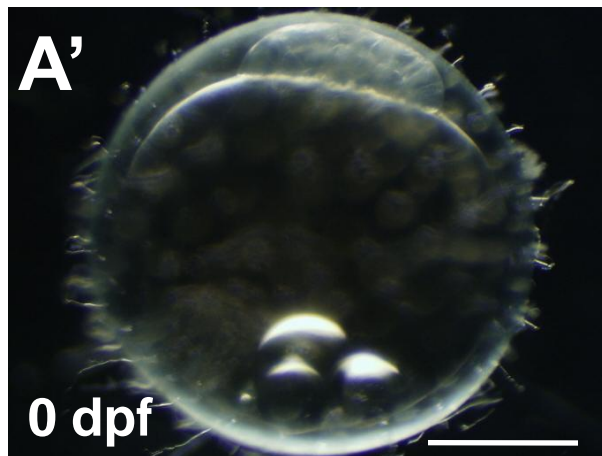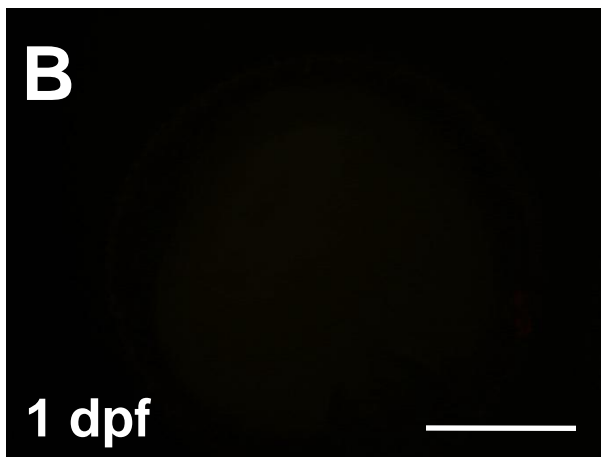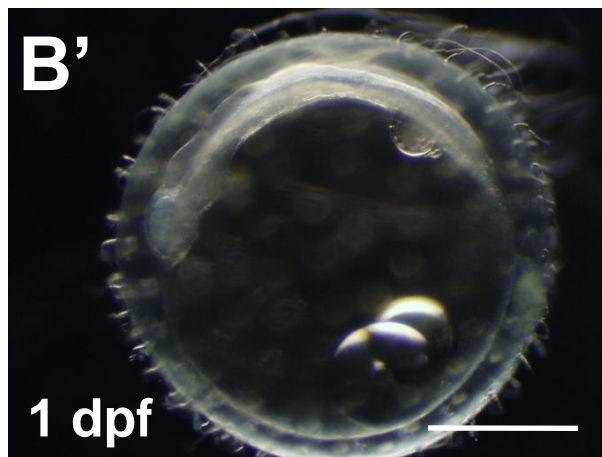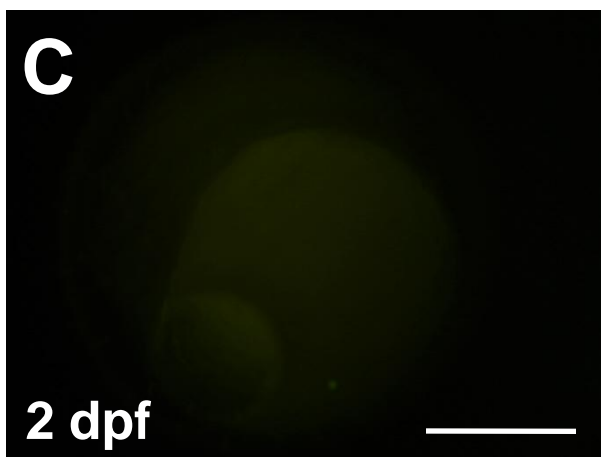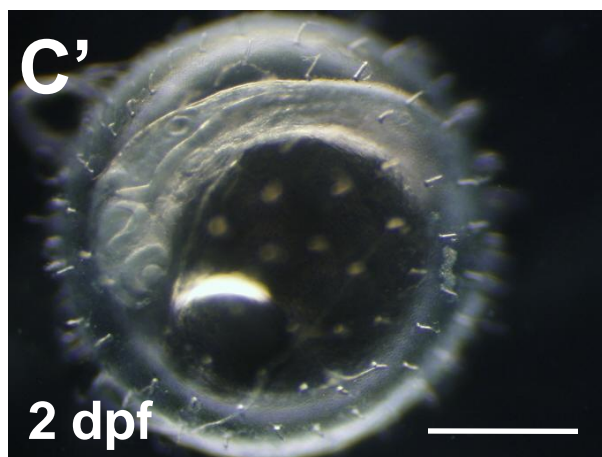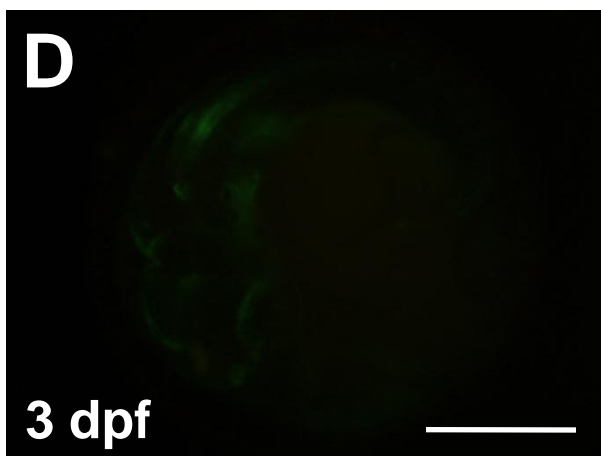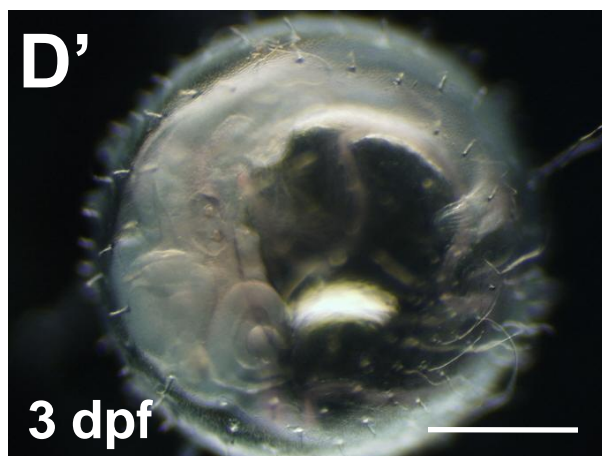

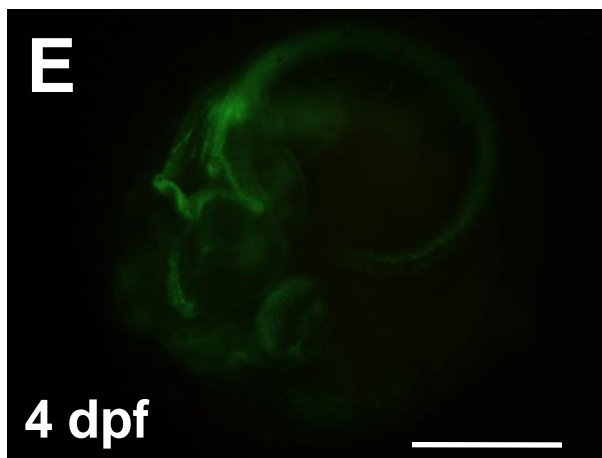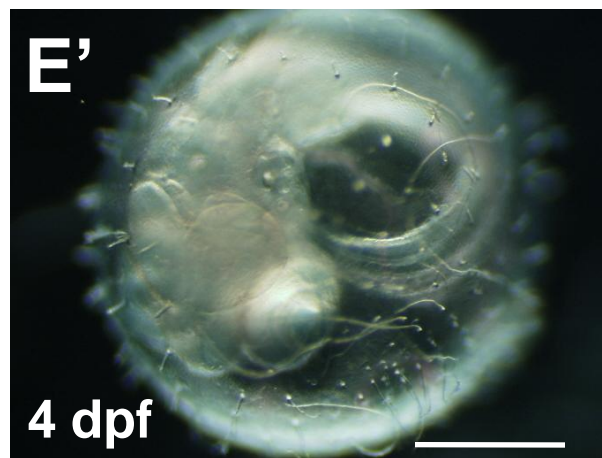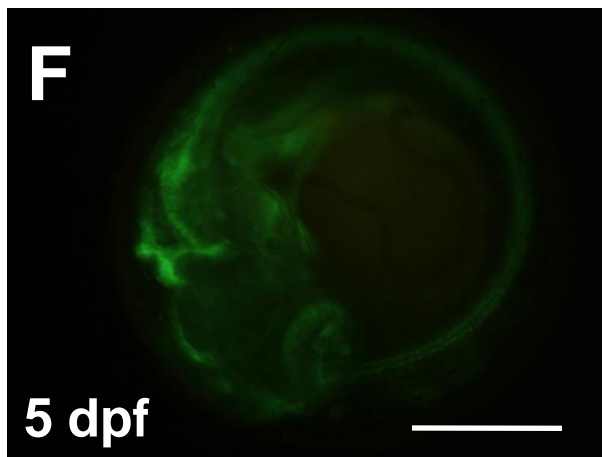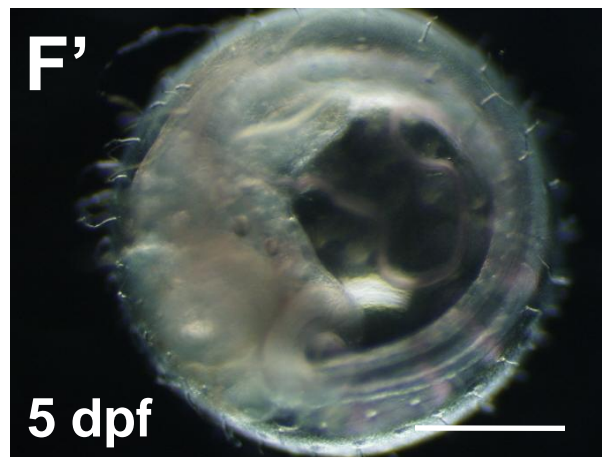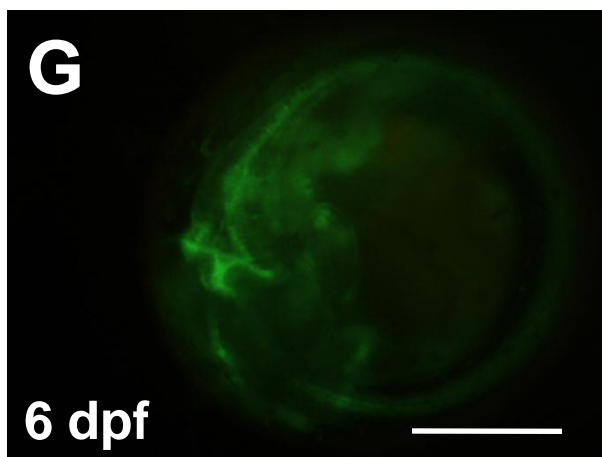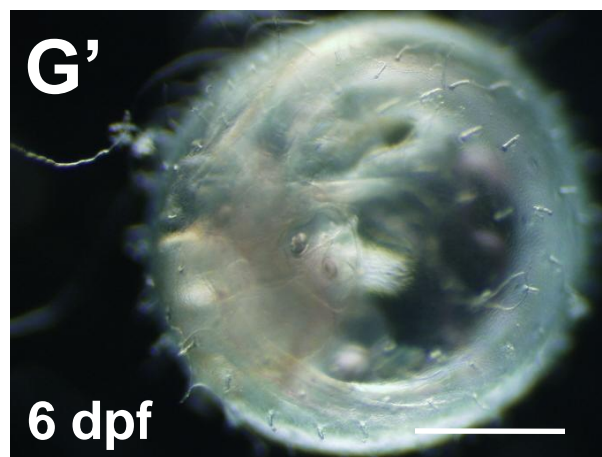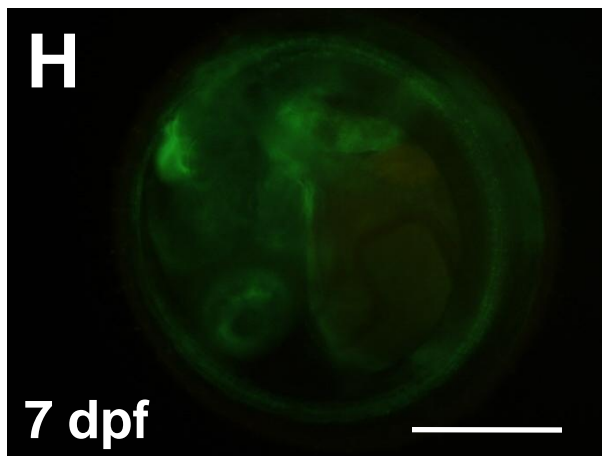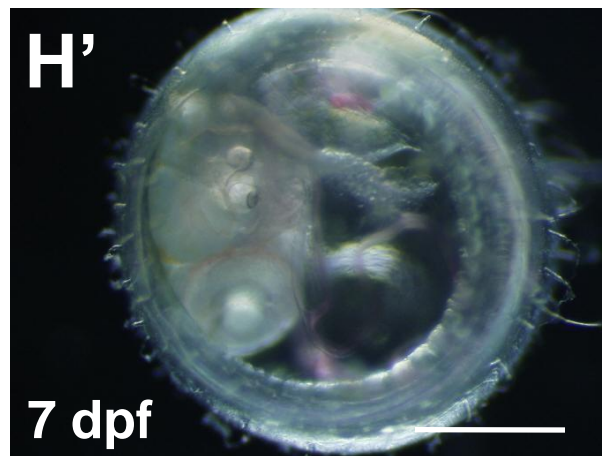

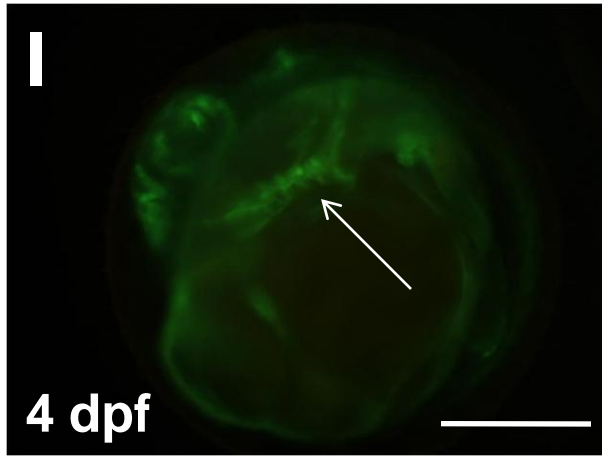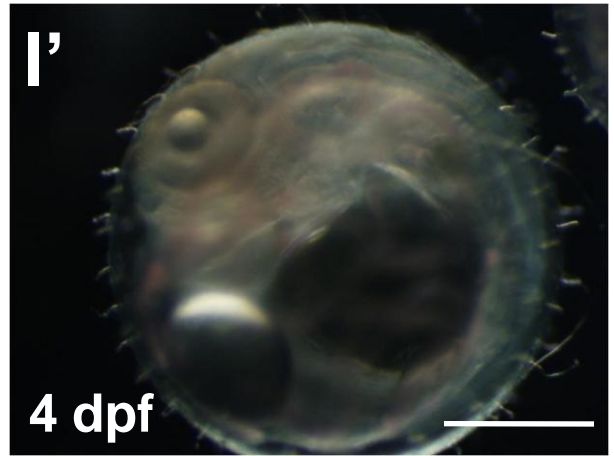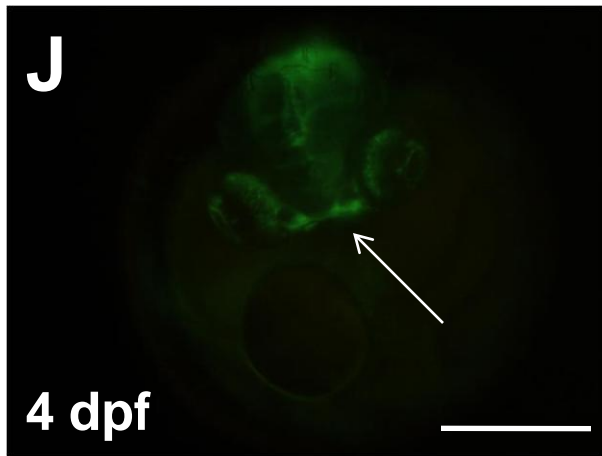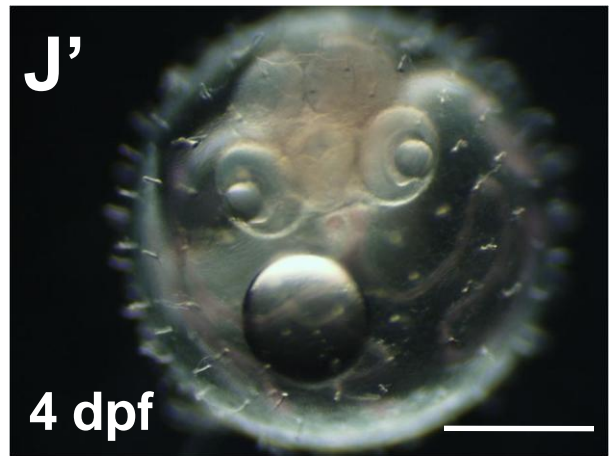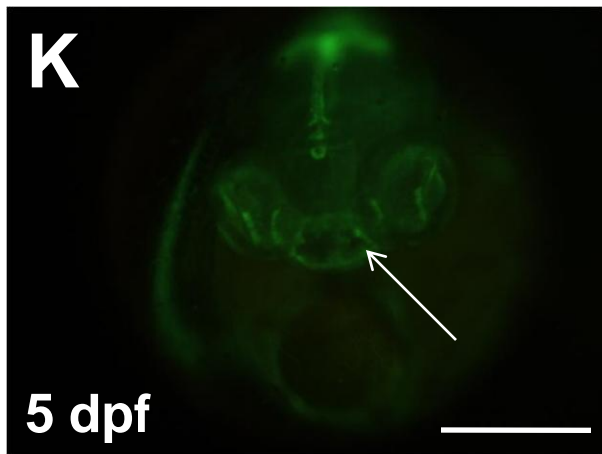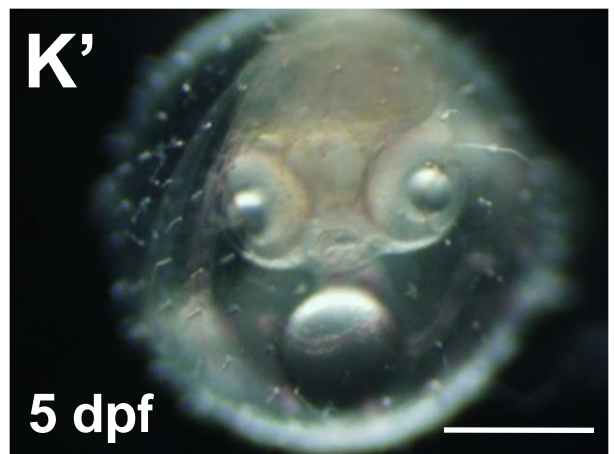

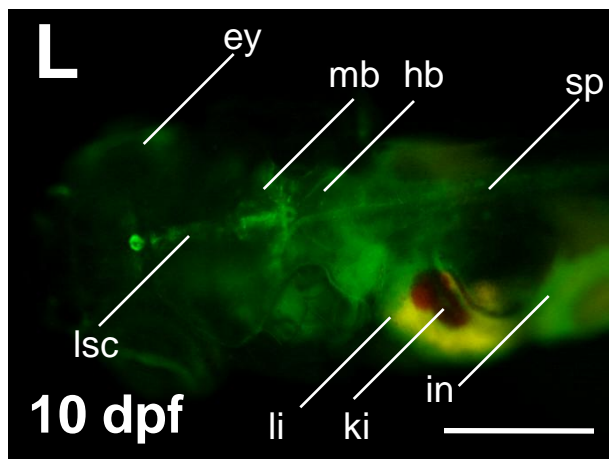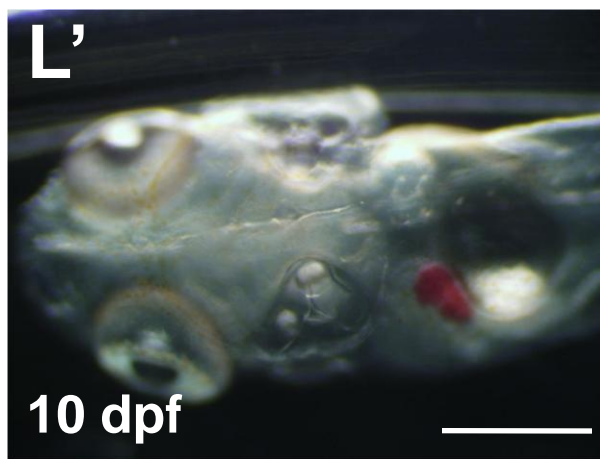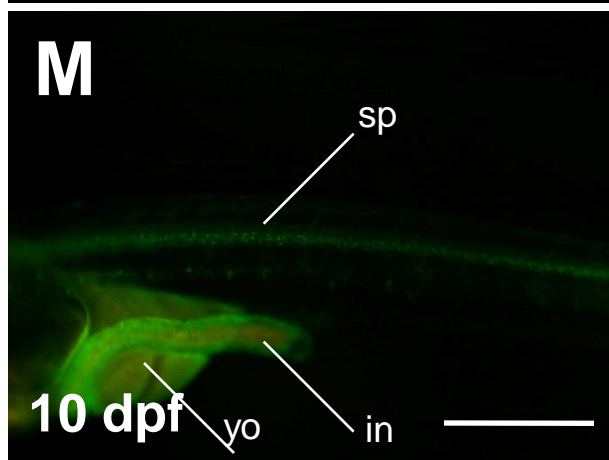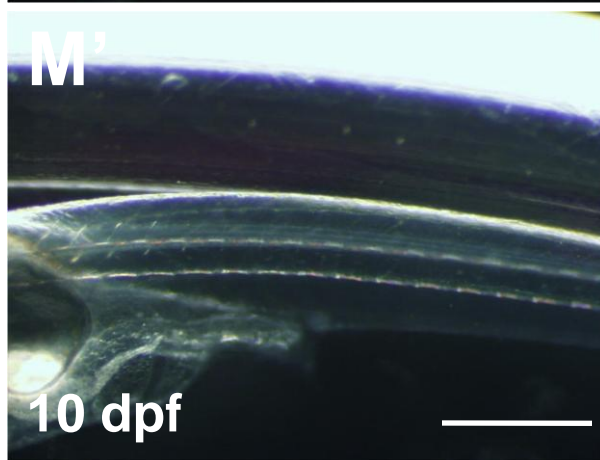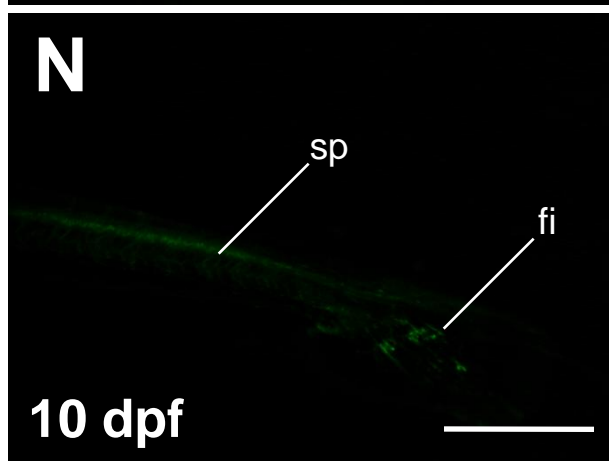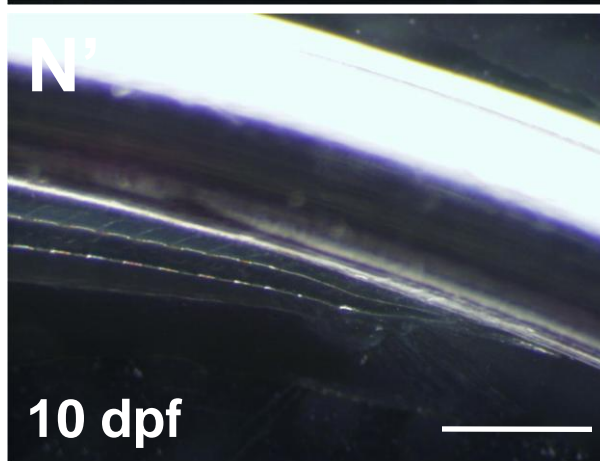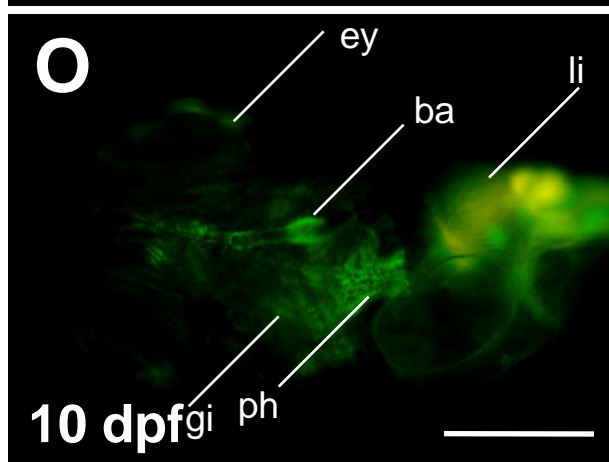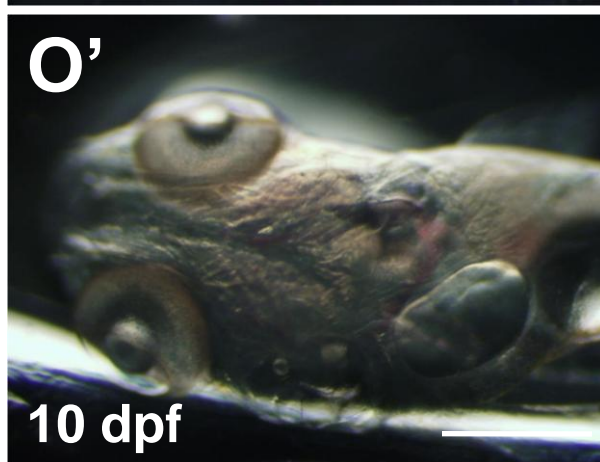

Supplement: Additional file 3 — GFP fluorescence of GFP fish. The pictures show fluorescent microscopy observations, A-O, and light microscopy, A'-O', of GFP fish. Embryonic stages, 0 dpf (A, A'), 1 dpf (B, B'), 2 dpf (C, C'), 3 dpf (D, D'), 4 dpf (E, I, J, E', I', J'), 5 dpf (F, K, F', K'), 6 dpf (G, G') and 7 dpf (H, H'), are shown. Distinct fluorescence was observed from 3 dpf. In embryonic stages, strong GFP fluorescence was observed in nervous system, branchial arches (I; white arrow) and olfactory epithelium (J, K; white arrow). Dorsal view of the anterior (L, L'), left side view of the middle, (M, M'), left side view of the tail (N, N'), and ventral view of the anterior (O, O') of 10 dpf are shown. ey: eye, mb: midbrain, hb: hindbrain, sp: spinal cord, lsc: longitudinal fissure of cerebrum, li: liver, ki: kidney, in: intestine, yo: yolk, fi: fin fibroblast, ba: bulbous arteriosus, gi: gill, ph: pharynx. Bars: 200 μm. [file 1471-2202-10-60-S3.pdf]
