# Supplementary material for: Functional and comparative genomics analyses of pmp22 in medaka fish
Source: BMC Neurosci. 2009 Jun 17;10:60. doi: 10.1186/1471-2202-10-60 (PMC2714311; doi:10.1186/1471-2202-10-60)
Supplement: Additional file 4 — Values of mRNA quantification of the total, 1A and 1B transcription levels. Total RNA was prepared from mixed extract of some fish at 4, 6, 8, 14 and 30 dpf. The upper table shows the threshold cycles of the beta-actin, internal control, and ol_pmp22 mRNA. The lower table shows the relative quantification of total, 1A and 1B transcript defining the average of each 4 dpf transcription level as 1.0. The graph is shown in Figure 6. The minimum and maximum were estimated according to the manufacturer's instructions (P < 0.05). [file 1471-2202-10-60-S4.pdf]

Threshold cycle of quantitative RT-PCR of *beta-actin* and *ol_pmp22* mRNA

	*beta-actin** (average Ct)	total *ol_pm22* (average Ct)	total *ol_pmp22* – *beta-actin* (Ct)	1A *ol_pm22* (average Ct)	1A *ol_pmp22* – *beta-actin* (Ct)	1B *ol_pm22* (average Ct)	1B *ol_pmp22* – *beta-actin* (Ct)
4 dpf	18.036	24.628	6.592	29.811	11.775	25.332	7.296
6 dpf	19.265	24.149	4.884	29.473	10.208	24.603	5.338
8 dpf	19.510	23.989	4.479	25.029	5.519	24.005	4.494
14 dpf	19.843	25.365	5.522	29.857	10.014	25.265	5.422
30 dpf	18.740	24.410	5.670	26.853	8.113	25.125	6.385

Ct: Threshold cycle

* internal control

Relative quantification of total, 1A and 1B mRNA levels

	total relative quantification* (Ct)	total Min.** (Ct)	total Max.** (Ct)	1A relative quantification* (Ct)	1A Min.** (Ct)	1A Max.** (Ct)	1B relative quantification* (Ct)	1B Min.** (Ct)	1B Max.** (Ct)
4 dpf	1.000	0.693	1.443	1.000	0.630	0.044	1.000	0.415	2.411
6 dpf	3.269	2.492	4.289	2.965	2.065	4.266	3.888	2.981	5.070
8 dpf	4.326	3.967	4.717	76.479	66.873	87.465	6.974	6.369	7.604
14 dpf	2.099	1.379	3.197	3.389	1.817	6.321	3.665	2.819	4.766
30 dpf	1.894	1.547	2.321	12.667	11.276	14.230	1.881	1.736	2.039

Ct: Threshold cycle

* relative quantification was calculated by dividing each level by corresponding 4 dpf level.

** Min. and Max. were estimated according to the manufacturer's instructions of Applied Biosystems.
